# *Plukenetia huayllabambana* Fruits: Analysis of Bioactive Compounds, Antibacterial Activity and Relative Action Mechanisms

**DOI:** 10.3390/plants9091111

**Published:** 2020-08-28

**Authors:** Armel Jackson Seukep, Minxia Fan, Satyajit Dey Sarker, Victor Kuete, Ming-Quan Guo

**Affiliations:** 1 CAS Key Laboratory of Plant Germplasm Enhancement and Specialty Agriculture, Wuhan Botanical Garden, Chinese Academy of Sciences, Wuhan 437004, China; seukep.armel@ubuea.cm (A.J.S.); fanminxia14@mails.ucas.ac.cn (M.F.); 2 Department of Biomedical Sciences, Faculty of Health Sciences, University of Buea, P.O. Box 63, Buea, Cameroon; 3 Sino-Africa Joint Research Center, Chinese Academy of Sciences, Wuhan 437004, China; 4 Innovation Academy for Drug Discovery and Development, Chinese Academy of Sciences, Shanghai 201203, China; 5 Centre for Natural Products Discovery, School of Pharmacy and Biomolecular Sciences, Liverpool John Moores University, Liverpool L3 3AF, UK; S.Sarker@ljmu.ac.uk; 6 Unit of Research in Microbiology and Antimicrobial Substances/Laboratory of Cancer Research, Department of Biochemistry, Faculty of Science, University of Dschang, P.O. Box 67, Dschang, Cameroon; victor.kuete@univ-dschang.org

**Keywords:** *Plukenetia huayllabambana*, solvent fractions, antibacterial, modes of action, GC–MS

## Abstract

*Plukenetia huayllabambana* is an edible plant traditionally used to cure wounds and various infections. The present work assessed, for the first time, the antibacterial efficacy of solvent fractions from *P. huayllabambana* fruits. The crude methanol extract was obtained applying ultrasound-assisted extraction, followed by partitioning through successive depletion among solvents of increasing polarity to yield fractions (n-hexane, dichloromethane, ethyl acetate, and n-butanol). The minimal inhibitory concentration (MIC) was determined following antibacterial testing, using the broth microdilution technique against a panel of drug-resistant Gram-negative and Gram-positive bacteria. Possible modes of action of the most active fraction were also investigated. Gas chromatography–mass spectrometry (GC–MS) was used to identify phytocompounds that may account for the recorded activities. Methanol, n-hexane (PH-n-Hex), and ethyl acetate extracts inhibited 100% of studied bacteria, with the recorded MIC ranging from 0.125–1 mg/mL. PH-n-Hex appeared as the most active partition, exerting a bacteriostatic effect. PH-n-Hex probably acts by interfering with bacterial biofilm formation, proton pumps, and bacterial cell membrane integrity. The GC–MS analysis of PH-n-Hex led to the identification of 11 potentially bioactive components, including fatty acids, phytosterol, and diterpene alcohol as major ones. *P. huayllabambana* can be considered as a plant of pharmacological value—a source of potent anti-infective drug entities.

## 1. Introduction

Antimicrobial resistance (AMR) is a rising threat to global health [1]. The AMR crisis leads to prolonged hospital stays, inflated medical costs, and a higher rate of death [1,2]. It is estimated that more than 700,000 people die of resistant infections every year. If no active solution to the problem is found now, the estimate says millions of people will die due to AMR in the coming years [3]. The scarcity of new, less toxic antibiotic molecules with a novel mode of action that are less prone to drug resistance propels the development of alternative medicine. Plants have been used since ancient times for the prevention and/or treatment of several disorders. The acquaintance of the plant healing properties has been inherited over the centuries within and among human communities [4,5]. Medicinal plants have important antibiotic biochemistry to protect themselves against various infections and these properties can be transferred to humans [4]. Indeed, plants produced active secondary metabolites that account for the pharmacological properties including the treatment of infectious diseases [6]. The data generated from the anti-infective efficacy of several herbals medicine used in traditional medicine have been scientifically confirmed [5,6,7,8,9,10,11,12,13]. Numerous studies have aimed to characterize the chemical composition of antimicrobials from plants, and their modes of action, either separately or in combination with commonly used antibiotics [5]. Edible plants are of growing interest nowadays [12,14]. The scientific validation and creation of research data on the quality, safety, and efficacy of folk medicine have become a pressing issue. Such scientific knowledge can help create an evidence-based traditional medicine that is increasingly respected by other public health professionals. The presence of phytochemicals (nutraceuticals), in addition to nutrients, in dietary fruits, spices, condiments, and other vegetables, is considered of crucial nutritional importance in the prevention of chronic diseases, including bacterial infections [12].

Plant species belonging to the family Euphorbiaceae contain a high diversity of phytochemicals, including terpenoids, phenolic compounds, alkaloids, and fatty acids [15,16]. This variability of plant chemicals may account for the varied uses of the plants from this family as among others, anti-infective agents [15]. The studied plant—*Plukenetia huayllabambana* (R. W. Bussmann, C. Téllez & A. Glenn) (Euphorbiaceae), also known as ‘Giant Sacha Inchi’ or ‘Giant Inca Peanut’, is a native plant to the tropical rain forest of the Amazon region of South America [17]. *P. huayllabambana* is closely related to another species of the same genus, namely *Plukenetia volubilis*, commonly known as ‘Sacha Inchi’ or ‘Inca Peanut’. The two species differ in morphological and physicochemical properties. The morphological dissimilarity between *P. volubilis* and *P. huayllabambana* might also involve variance in the phytochemical composition. Indeed, Chirinos and collaborators [18] revealed pretty similarly fatty acid profiles for both species but *P. huayllabambana* presented a significantly higher content of alpha-linolenic acid than *P. volubilis*, while important contents of γ- and δ-tocopherol were evidenced in both oils. β-Sitosterol was the most important and representative phytosterol. In Cameroon, *P. huayllabambana* is used for alimentation and therapeutics. Seeds are edible (in a cooked or roasted form), while other parts of the plant are used traditionally to cure wounds and various infections. Most of the reported biological effects are related to seed-derived oils, while there are almost no studies on organic extracts. Sacha inchi oil-related products have been reported to exhibit anti-adherent activity against *Staphylococcus aureus*, skin tightening, anti-aging effects [19], and anti-inflammatory properties [20]. *P. huayllabambana* has been characterized as a rich source of fatty acids (saturated, mono- and polyunsaturated) comprising linolenic and linoleic acids as major ones. It also contains varied pharmacologically active compounds such as γ- and δ-tocopherol, phytosterol (high content of sitosterol), phenyl alcohols, and phenolic compounds including flavonoids, secoiridoids, and lignans [18,21,22]. In the present work, we evaluated the antibacterial efficacy of the crude methanolic extract and solvent fractions from *P. huayllabambana* fruits against five drug-resistant Gram-negative and Gram-positive bacteria, and the evaluation of the antibacterial modes of action. The gas chromatography coupled to mass spectrometry (GC–MS) analysis for bioactive components identification was performed on the n-hexane fraction.

## 2. Results and Discussion

### 2.1. GC–MS Profiling of Potential Bioactive Phytoconstituents

**Figure 1:**
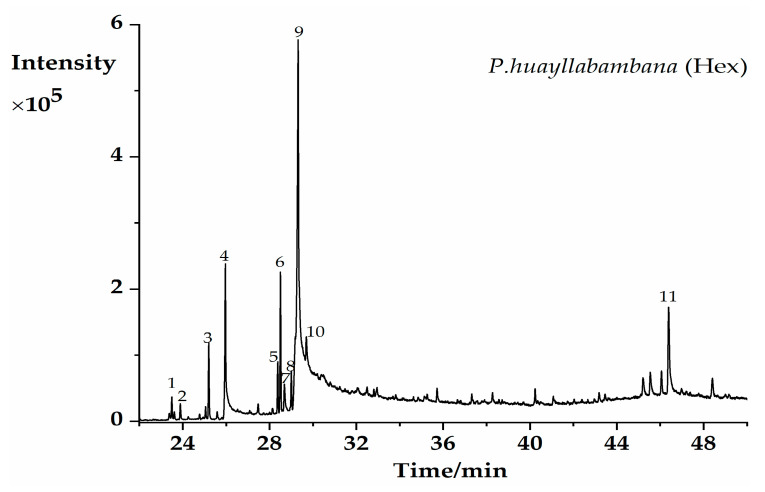
Gas chromatography–mass spectrometry (GC–MS) chromatogram of the *P. huayllabambana* n-hexane fraction. The peak numbers in this figure correspond to those used in Table 1.

**Figure 2:**
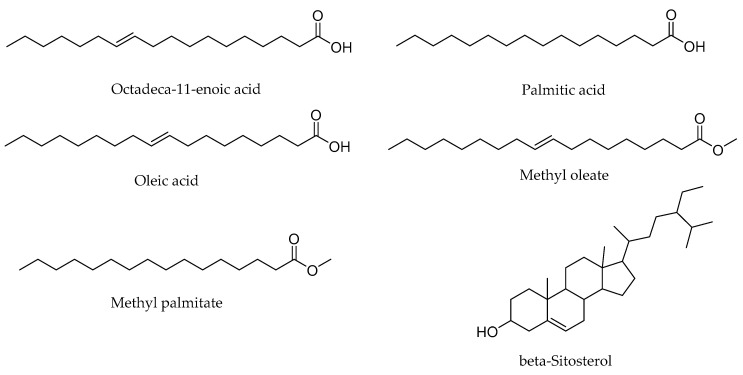
Chemical structures of identified major bioactive components from the n-hexane fraction of *P. huayllabambana*.

The GC–MS analysis of PH-n-Hex led to the identification of 11 phytoconstituents (Figure 1, Table 1). Only compounds that showed the significance of similarity percentage ≥ 85% were considered. Above this value, there is a high probability to get a correct identification [23]. Major components identified were monounsaturated (oleic acid, octadeca-11-enoic acid, and methyl oleate,) and saturated (palmitic acid, methyl palmitate) fatty acids and beta-sitosterol. Other identified compounds with an area percentage < 2% included methyl linoleate, phytol, methyl stearate, hexahydrofarnesyl acetone, and diisobutyl phthalate. Oleic acid [24] was identified as the main component (46.55%). Our results were consistent with previous findings, since some studies displayed similar phytochemicals constituents from *P. huayllabambana*, in particular, the fatty acid and phytosterol contents [18,25]. Indeed, the investigations carried out by Ramos-Escudero et al. [25] reported the presence of oleic acid, palmitic acid, and triterpene alcohol in *P. huayllabambana*. Chirinos et al. [18] previously identified β-sitosterol as the most important and representative phytosterol (as in most plants) found in *P. huayllabambana*. The chemical structures of the major phytoconstituents identified were shown in Figure 2. 

### 2.2. Antibacterial Activity

The upsurge of bacterial drug-resistant infections is a permanent public health concern. The resistance crisis urges the scientific community to seek for effective alternative therapeutic compounds with unusual modes of action [1]. This accounts for the rush for natural substances comprising medicinal plants. Indeed, medicinal plants produce structurally diverse secondary metabolites with a wide range of biological properties [26]. In the present study, we examined the antibacterial efficacy of the methanol extract (PH-MeOH) and subsequent partition fractions (n-hexane, dichloromethane, ethyl acetate, and n-butanol) from *P. huayllabambana* fruits. The recorded minimal inhibitory concentration (MIC) values are summarized in Table 2. Bacteria used in this work (*Enterococcus faecalis*, *Staphylococcus aureus*, *Salmonella enterica*, *Klebsiella pneumoniae*, and *Enterobacter cloacae)* are among those prioritized by the World Health Organization (WHO) in the research for novel antibacterial agents [1]. Preliminary investigations on the antibiotic resistance profile confirmed the resistance features of studied bacteria (data not shown). The studied Gram-positive and Gram-negative bacteria were found to be sensitive to the test solvent fractions. The latter were selectively active, with MIC values ranging from 0.125–1 mg/mL (Table 2). No bactericidal effects were recorded. The variation in activity recorded was probably linked with the type of extraction solvent. This may indicate that some antimicrobial active principles in *P. huayllabambana* fruits dissolve in varying degrees in solvents used. PH-MeOH, PH-n-Hex, and the ethyl acetate fraction (PH-EA) displayed noteworthy inhibitory effects, preventing the growth of all tested bacterial strains (100%). 

Except for the n-butanol fraction (PH-n-BuOH), other fractions showed potential strong activities (MIC ≤ 0.5 mg/mL) against *S. enterica*. The antibacterial activity of plant extracts is considered very interesting (highly active) if MIC < 0.1 mg/mL, active or moderately active if 0.1 ≤ MIC ≤ 1 mg/mL, and low if MIC > 1 mg/mL [27]. Therefore, no highly active extract was obtained from the present study. However, since recorded MICs varying from 0.125 to 1 mg/mL, these suggested moderate activity against studied drug-resistant bacteria. The best activities were obtained with PH-MeOH and PH-n-hexane against *S. enterica*, and the two Gram-positive bacteria *E. faecalis* and *S. aureus*. To the best of our knowledge, the antibacterial activity of PH-MeOH and subsequent partition fractions from the fruits of *P. huayllabambana* (Euphorbiaceae) is being reported in this study for the first time. However, the antibacterial properties of many plants of the family Euphorbiaceae have previously been reported in the literature [15,16,28]. Few studies have documented the antimicrobial properties of plants from the genus *Plukenetia*. The present investigation, therefore, provides valuable information for the antibacterial activity of *P. huayllabambana*. The antibacterial activity of *P. huayllabambana* solvent fractions was probably due to the presence of naturally occurring bioactive components. The nature of the solvent of extraction, the amount of various chemical components in the extract, as well as the possible interactions between them, are some factors that can influence the activity. The differences in sensitivity recorded for the same extract with different bacteria strains could be explained by intrinsic differences in the chemical composition of the bacterial cell wall and/or in the genetically resistant elements, which may/not be transferable between strains such as plasmids or transposons [29]. Moreover, the differences recorded for the same bacterium and different fractions can be due to the qualitative and quantitative differences of antimicrobial active principles or by differences in the mechanisms of action of bioactive constituents [30]. Indeed, the active ingredients targeting the bacterial cell wall must find complementary receptors for their interaction that are appropriate for their action, while those acting inside the cell must be both able to cross the membrane and find target elements in the cell [14]. Among the five bacteria tested, Gram-negative bacteria (*E. cloacae* and *K. pneumoniae*) were found to most resist the effects of the test samples. These results were consistent with the common observations that Gram-negative bacteria are usually more resistant to botanicals than their counterpart Gram-positive ones. The resistance of Gram-negative bacteria towards antibacterial substances was owed to the presence of an outer membrane that takes part in the natural resistance by acting as an effective permeability barrier [31]. Common biological effects, comprising antibacterial activities, of *P. huayllabambana* were related to derived oils, commonly known as sacha inchi oils. For instance, sacha inchi oil-related products have been reported to exhibit anti-adherent activity against *S. aureus* [18], anti-aging effects [19], and anti-inflammatory properties [20]. The fact that PH-n-Hex was found to be the most effective fraction could probably be linked to its oil extraction capacity. Accordingly, in addition to its notable antibacterial effects, we have selected the lipophilic fraction (PH-n-Hex) to evaluate the possible mechanisms of action, as well as the identification of potentially bioactive components via GC–MS analysis. 

The antimicrobial activities of the major compounds identified were widely documented. The possibility of the therapeutic use of fatty acids (both saturated and unsaturated) as antimicrobial agents is worthy of note [32], and the main ones identified in the present study could explain the antibacterial potential exerted by PH-n-Hex. Octadeca-11-enoic acid, an omega-7 fatty acid, is well known for its antibacterial activity [33]. Previous investigations also reported the antimicrobial activity of linoleic acid (and derivatives) and oleic acid against Gram-positive bacteria [34]. Likewise, palmitic acid, a long-chain saturated fatty acid, has been reported to exert notable antimicrobial activities against oral pathogens (bacteria and fungi) [35]. Furthermore, Sharma [36] underscored the antimicrobial activities of plant sterols including the one identified in the present study (beta-sitosterol). In addition, Sen and co-workers [37] have reported significant antimicrobial activities of beta-sitosterol against Gram-negative and Gram-positive bacteria including *S. aureus* and *K. pneumoniae*, some of the bacteria species used in this work. A diterpene alcohol phytol identified has been also reported to act as an antimicrobial agent [38]. The presence of these compounds in *P. huayllabambana* makes it a plant of pharmaceutical value. The identified compounds could act alone, or in interactions with other constituents of the mixture. The present work provided evidence of the traditional use of *P. huayllabambana* as an anti-infective plant. Most of the bacteria species examined in the present study are implicated in gastrointestinal troubles. Therefore, various solvent extracts from *P. huayllabambana* fruits, particularly PH-n-Hex, can be considered as promising candidates in the control of gastrointestinal diseases.

### 2.3. Antibacterial Action Mechanisms

#### 2.3.1. Antimicrobial Efficacy Testing

**Figure 3:**
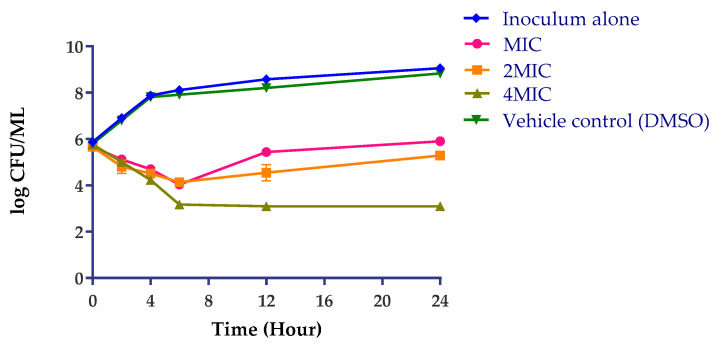
Antibacterial efficacy testing of *P. huayllabambana* n-hexane fraction on *S. enterica*. Time intervals: 0, 2, 4, 6, 12, and 24 h. Data are expressed as mean ± SD (*p* < 0.05). MIC: minimum inhibitory concentration.

Due to their chemical and structural diversity, plant-derived components act on different targets in bacteria cell structure including membrane, cell wall, and/or on molecular targets (ions or protons, proteins, DNA/RNA) by several mechanisms of action [14]. The antibacterial modes of action of PH-n-Hex were investigated against *S. enterica*. That fraction was tested for antibacterial efficacy over time (24 h) at different concentrations (MIC, 2MIC, and 4MIC). At all tested concentrations (Figure 3), PH-n-Hex decreased bacterial counts over 24 h when compared to the starting inoculum. With increasing sample concentrations, a concentration-dependent trend towards greater bacterial killing was observed. The overall effect was bacteriostatic: PH-n-Hex inhibited the growth and reproduction of bacteria but did not kill them. This may point out the effect on bacterial metabolism. Like any bacteriostatic agent, a clinical application would require a collaboration of the bioactive agents with the host immune system to oust pathogenic bacteria from the body. However, high concentrations (>4MIC) could induce bactericidal effects.

#### 2.3.2. Action on the Cell Membrane

**Figure 4:**
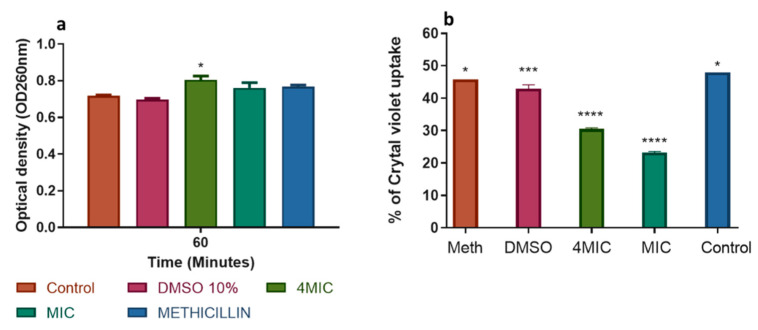
Modes of action of *P. huayllabambana* n-hexane fraction on *S. enterica* cell membrane. (**a**) Action on cell membrane integrity. Absorbance measurement of intracellular components (DNA, RNA) at OD260 nm after 1 h of incubation. (**b**) Action on membrane permeability. All data are expressed as mean ± SD (*p* < 0.05). Meth: methicillin. MIC: minimum inhibitory concentration.

The action of the test extract (PH-n-Hex) on the *S. enterica* cell membrane was shown in Figure 4. The quantification of the release of UV-absorbing materials at OD260 was an indication of a serious and irreversible alteration of the cytoplasmic membrane. The release of intracellular content was an index of damage and loss of membrane integrity [39]. After treatment with PH-n-Hex at MIC and 4MIC, the OD increased up to 0.803 from 0.71 (Figure 4a). These observations implied that the test sample weakly damages the cytoplasmic membrane thereby leading to low leakage of *S. enterica* intracellular constituents. The percentage of crystal violet uptake significantly decreased after treatment with test material at MIC and 4MIC compared to untreated cells (Figure 4b). The difference in uptake of crystal violet from the bacterial cell wall after treatment compared to normal could be due to defective cell wall, as there were permeability changes [39]. The reduction in crystal violet uptake could probably be attributed to the enlargement of the treated cell, thereby changing the permeability and structure of the cell wall membrane layer compared to normal cells.

#### 2.3.3. Action on H^+^-ATPase-Mediated Proton Pumping

**Figure 5:**
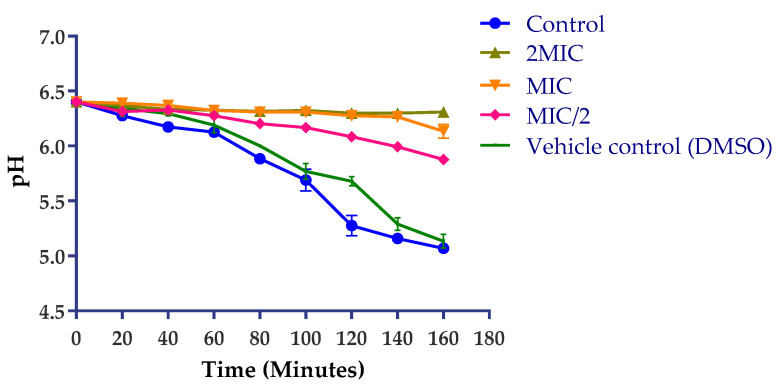
Action *P. huayllabambana* n-hexane fraction on *S. enterica* H^+^-ATPase-mediated proton pumping as a function of time. MIC: minimum inhibitory concentration. Data expressed as mean ± SD (*p* < 0.05).

The inhibition of *S. enterica* H^+^-ATPase-mediated proton pumping was noted after treatment with PH-n-Hex (Figure 5). Ion exchange systems in bacteria are coupled to the ATP energy synthesis used by the bacteria and any inhibition of their functioning (which results in a reduction in the acidification of the medium) could be detrimental to their survival. Cytoplasmic pH of bacteria cells is regulated by extrusion proton through the respiratory chain and K^+^ influx at acid pH, and cation/proton antiporter regulates the pH in alkaline states [40]. Every substance that disturbs the regulation of ATPase responsible for maintaining the homeostasis and osmotic stability of ions inside the cell is considered as a target of proton pumps. As shown in Figure 5, the test fraction induced inhibition of the glucose-induced acidification of the external medium by *S. enterica* in a time- and concentration-dependent manner. For instance, at 2MIC, the extract exerted a constant and total inhibition of *S. enterica* proton pumps, suggesting that the H^+^-ATPase of *S. enterica* is a potential cellular target of PH-n-Hex.

#### 2.3.4. Action on Biofilm Formation

**Figure 6:**
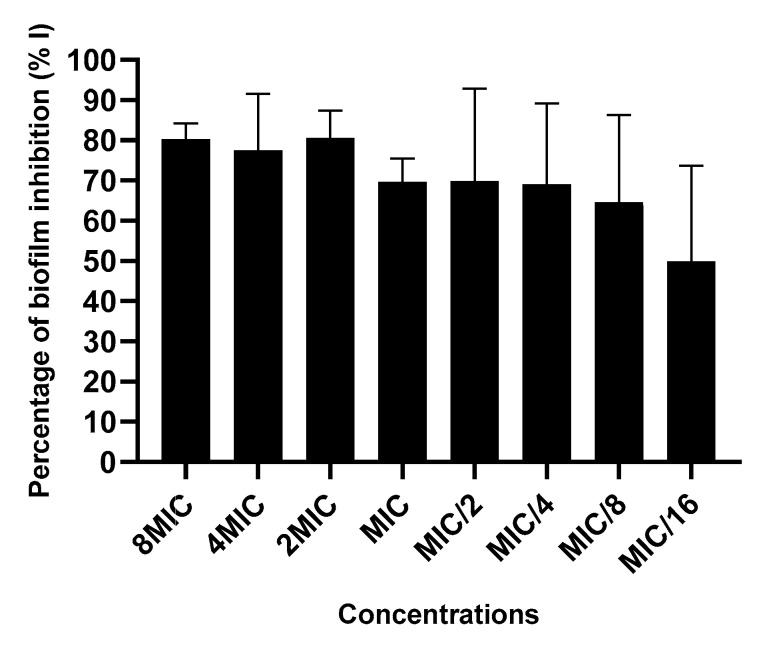
Action of *P. huayllabambana* n-hexane fraction on *S. enterica* biofilm formation. MIC: minimum inhibitory concentration. Data expressed as mean ± SD (*p* < 0.05).

PH-n-Hex depicted strong inhibition of *S. enterica* biofilm formation (up to 80%) at inhibitory concentrations (MIC, 2MIC, 4MIC, 8MIC) and between 50% and 70% at sub-inhibitory concentrations (MIC/2, MIC/4, MIC/8, MIC/16) (Figure 6). No significant difference (*p* < 0.05) has been found between the values. The inhibition of the biofilm formation recorded at sub-inhibitory doses indicates the anti-biofilm activity of PH-n-Hex phytoconstituents. The molecular mechanism of plant extract on biofilm structure consists of targeting peptidoglycan synthesis and modulating the quorum sensing (QS), a whole gene involving in the regulation of biofilm formation [41]. Indeed, the QS regulates various functions in bacteria, including biofilm formation, antibiotic resistance, as well as the ability of bacteria to induce disease. The inhibition of biofilm formation by PH-n-Hex may be assigned to the presence of pharmacologically active phytoconstituents that act specifically on QS signaling pathways. Thereby, plant extract and its active compounds could inhibit signaling pathways of the QS that are involved in biofilm formation [42].

## 3. Materials and Methods

### 3.1. Chemicals and Reagents

Bacterial culture media, trypticase soy agar (TSA) and trypticase soy broth (TSB), were purchased from Qingdao Hope Bio-Technology (Qingdao, China). Para-Iodonitrotetrazolium chloride (INT) 98% was provided by Macklin (Shanghai, China). Dimethyl sulfoxide (DMSO ≥ 99.0%) was obtained from Sinopharm Chemical Reagent (Shanghai, China). Streptomycin (purity > 98%) (Abmole Biosciences, Houston, TX, USA) and methicillin (purity > 98%, Cayman Chemical, Ann Arbor, MI, USA) were used as reference antibiotics for antibacterial testing.

### 3.2. Plant Material 

Fruits of *P. huayllabambana* were collected in Bangangté, West Region, Cameroon (coordinates: 5.0880° N, 10.5184° E) in July 2018. The authentication of the plant sample was done at the National Herbarium of Cameroon (HNC, Yaoundé, Cameroon) under a reference number (590718HNC). The fruits were cleaned out thoroughly and then cut into small pieces and air-dried, away from direct sunlight for 2 weeks. Afterwards, the air-dried plant material was ground into a fine homogenous powder. The powder obtained was packaged and kept at room temperature for future use. 

### 3.3. Extraction Process of Plant Material

The air-dried plant powder (100 g) was macerated into methanol (1:10 *w*/*v*) for 24 h. After that, ultrasound-assisted extraction (KQ-500DE, Kunshan Ultrasonic Instrument Co., Ltd., Kunshan, China) was performed on the macerate for 30 min, followed by filtration using Whatman filter paper grade 1. The same procedure was repeated twice with the remaining residue. The overall filtrate was concentrated in a rotary evaporator at reducing pressure and temperature (<45 °C) to afford 7.68 g of crude methanolic (MeOH) extract. Partitioning was prosecuted following the protocol previously described by Parsaee et al. [43]. Briefly, the resulting MeOH extract was first suspended in distilled water. Then, the aqueous solution obtained was partitioned by successive depletion among solvents of increasing polarity including n-hexane, dichloromethane, ethyl acetate, n-butanol, and finally water. Dried extracts were obtained after evaporation of residual solvents to yield residues of 1.8 g, 0.3 g, 0.4 g, 0.5 g, and 1.6 g, respectively. MeOH extract and fractions were kept at 4 °C until further use.

### 3.4. Gas Chromatography-Mass Spectrometry (GC–MS) Analysis

The GC–MS analysis of the n-hexane fraction (PH-n-Hex) was performed using an Agilent 7890A GC–MS system, operating in an electron-impact mode of 70 eV. The GC system was equipped with a capillary column Agilent 19091S-433 (325 °C, 30 m long × 250 µm id × 0.25 µm film thickness) (Agilent Co., Santa Clara, CA, USA). The chromatographic conditions were as follows: sample preparation in hexane; injection volume 1 µL; split ratio 1:10; carrier gas helium flow rate 1.0 mL/min; column oven temperature programmed from 60 to 300 °C, with a gradient of 5 °C/min; injector temperature 250 °C. The identification of the chemical components was done by comparing their mass spectra and retention time with the data available in the National Institute of Standards and Technology (NIST) library (GC–MS System Nist. 11 lib.). 

### 3.5. Bacteria Strains

Studied bacteria strains were from CCTCC (China Center for Type Culture Collection), CMCC (Center for Medical Culture Collection), and ATCC (American Type Culture Collection). These comprised two Gram-positive (*Staphylococcus aureus* CCTCC AB91093 and *Enterococcus faecalis* ATCC29212) and three Gram-negative bacterial strains (*Salmonella enterica* CCTCC AB94018, *Enterobacter cloacae* ATCC700323, and *Klebsiella pneumoniae* CMCC(B)46117). Trypticase soy agar (TSA) and trypticase soy broth (TSB) were used as culture media for antibacterial testing. TSA and TSB are non-selective, general-use culture media that supply enough nutritive elements, allowing for a broad variety of microorganisms to grow. Before any experiment, studied bacteria were subcultured (at 37 °C for 18–24 h) in TSA. TSB was used for microdilution and antibacterial mechanistic studies. Bacteria inoculum was initially prepared in sterile distilled water, the turbidity adjusted with a spectrophotometer to a McFarland standard of 0.5, equivalent to 1.5 × 10^8^ CFU/mL. 

### 3.6. INT Colorimetric Assay for MIC and MBC Determination

The antibacterial assay of MeOH extract and fractions were carried out in triplicate using a broth microdilution method for plant extracts in 96-wells microplates, using INT as the bacterial growth indicator [7,8,9,10,11,13,44]. Test extracts and reference antibiotics (Methicillin and Streptomycin) were dissolved in DMSO/TSB to obtain the working solution. The final concentration of DMSO in the assay was less than 2.5%, a concentration innocuous to bacterial growth. The solution obtained was added to TSB, followed by a two-fold serial dilution in a 96-wells microplate. Subsequently, bacterial suspension initially prepared at the McFarland standard of 0.5 (1.5 × 10^8^ CFU/mL), as mentioned, was diluted in TSB, and 100 μL of bacterial inoculum was seeded in the wells of plates containing test extracts. The final inoculum concentration was equivalent to 1.5 × 10^6^ CFU/mL. Afterwards, the plates were covered with a sterile plate sealer, then shaken to mix the contents of the wells using a plate shaker, followed by incubation at 37 °C for 18 h. Wells containing adequate TSB, 100 μL of inoculum, and DMSO to a final concentration of 2.5% served as negative controls. The final concentration of the extracts varied from 0.008 to 1 mg/mL, whereas that of antibiotics ranged from 0.002 to 0.25 mg/mL. The minimal inhibitory concentration (MIC) of test samples was recorded after 18 h incubation at 37 °C, following the addition (40 μL) of INT 0.02% (*w*/*v*) and another incubation at 37 °C for 30 min. The viable bacteria reduced the yellow dye to pink. Wells with test samples only (blank control) were used to ensure that the extracts were not producing color with INT. The minimal bactericidal concentration (MBC) was assessed by adding 50 μL aliquots of the preparations, which did not show any growth after incubation during MIC testing, to 150 μL of adequate broth. These preparations were incubated at 37 °C for 48 h. The MBC was considered as the lowest concentration of samples that prevented the color change of the medium after the addition of INT as abovementioned. The assays were performed in triplicate and repeated thrice. 

### 3.7. Action Mechanisms

The n-hexane fraction (PH-n-Hex) appeared as the most active among partitions obtained, after the successive depletion of methanol extract. That fraction was used for mechanistic studies against the most sensitive bacteria strain (*S. enterica*). 

#### 3.7.1. Antimicrobial Efficacy Testing

The antimicrobial efficacy assay (time-kill kinetic assay) is applied to evaluate the activity of an antimicrobial agent against a bacterial strain and can establish the bactericidal or bacteriostatic activity of a compound over time. The assay was conducted based on the protocol earlier described by Appiah et al. [45], with slight modifications (final concentrations of test extract were equal to 4MIC, 2MIC, and MIC, whilst the time intervals for the kinetic study was as follows: 0, 2, 4, 6, 12, and 24 h). The inoculum concentration was equivalent to 1.5 × 10^8^ CFU/mL. 

#### 3.7.2. Action on Cell Membrane Integrity: Measurement of Intracellular Components (DNA/RNA)

The release of UV-absorbing material concentrations was measured by UV–VIS spectrophotometer following the study conducted by Devi et al. [39]. Briefly, overnight cultures of *S. enterica* in TSB were adjusted to achieve the OD600 of 2.0. Cells were harvested by centrifugation (400× *g*, 15 min), the supernatant was discarded, and the pellet was washed twice (using sterile deionized water) and then suspended in phosphate buffer saline (PBS, pH 7.4). Different concentrations of extract corresponding to MIC and 4MIC were added to the cell suspension. Methicillin was used as a positive control and cells without extract treatment were used as negative controls. The experiment was done in triplicate. All the samples were incubated at 37 °C for 60 min under agitation. After treatment, the cell suspension was centrifuged (13,400× *g*, 15 min) and the OD260 value of the supernatant was taken as a percentage of the extracellular UV-absorbing materials released by cells. All the measurements were done in triplicates in MAPADA UV–VIS 1100 spectrophotometer (Shanghai Mapada Instruments Co., Ltd., Shanghai, China).

#### 3.7.3. Action on Membrane Permeability

Crystal violet assay was applied to investigate the alteration in membrane permeability according to the protocol described by Devi et al. [39]. Briefly, suspensions of *S. enterica* were prepared in TSB. Cells were harvested after centrifugation (4500× *g*, 5 min, 4 °C) and were washed twice and suspended in PBS (pH 7.4). The bacteria cell suspension in buffer was treated with PH-n-Hex (at MIC and 4MIC) and methicillin (at MIC) followed by incubation (37 °C, 30 min). Likewise, control samples were prepared similarly without treatment. Another centrifugation (9300× *g*, 5 min) allowed the harvesting of cells, followed by the re-suspension in PBS containing 10 µg/mL of crystal violet. Afterward, the solution was incubated for 10 min at 37 °C, followed by centrifugation (13,400× *g*, 15 min), and the OD590 of the supernatant was measured using MAPADA UV–VIS 1100 spectrophotometer. The OD value of the crystal violet solution, which was originally used in the assay, was taken and it was considered as 100%. The percentage of crystal violet (CV) uptake of tested samples was calculated using the following formula (Equation (1)):

% of CV uptake = [OD value of the sample/OD value of crystal violet solution] × 100(1)

#### 3.7.4. Action on H^+^-ATPase-Mediated Proton Pumping 

The ability of PH-n-Hex to inhibit the *S. enterica* H^+^-ATPase-mediated proton pumping was assessed by monitoring the acidification of the external medium through pH measurement using a pH-meter equipped with E-201-C combined electrode (PHSJ-3F pH Meter, INESA Scientific Instrument Co., Ltd., Shanghai, China), based on previously described protocol [28,46]. Briefly, 100 mL bacteria culture (1:100 *v*/*v*) was grown in TSB culture medium for 18 h at 37 °C, followed by centrifugation (3500× *g*, 10 min). The pellet was washed twice with distilled water and then with 50 mM KCl and re-suspended in 50 mL of 50 mM KCl. The cell suspension (1.5–2 × 10^8^ CFU/mL) was incubated overnight (18 h, 4 °C) for glucose starvation and then centrifuged. In 4 mL of the reaction medium, 0.5 mL of PH-n-Hex corresponding to MIC/2, MIC, and 2MIC was added and the pH adjusted to 6.4. Upon 10 min pre-incubation under agitation at 37 °C, the acidification of the medium was initiated following the addition of 0.5 mL of glucose 20% (*w*/*v*). The pH measurement was recorded every 20 min for 160 min. The experiment was conducted in the presence of DMSO (control) at a final concentration of 2.5%, to measure the extent of acidification of the external medium in the absence of the plant extract. The experiment was performed in triplicate and repeated twice. The measured pH values were used to plot the pH evolution curve as a function of time [pH = f (time)]. Any inhibition of the acidification of the medium in the presence of the extract has been ascribed to an inhibitory effect of the H^+^-ATPase pumps.

#### 3.7.5. Action on Biofilm Formation 

Biofilm assay was assessed using crystal violet assay (96-well microtitre plate assay for biofilm quantification), as previously described [47,48]. The first phase of the assay was similar to that of MIC determination, mentioned above. The serial twofold dilution led to the concentration ranging from 1/16 MIC to 8 MIC. However, the incubation time was 24 h at 37 °C. The next step consisted of gentle removal of each well content by tapping the plates. Subsequently, the wells were washed with 200 µL sterile saline solution (NaCl 0.9%) to remove free-floating bacteria and then dried and fixed at 65 °C for 1 h. Biofilms formed by adherent cells in plates were stained with 0.8% (*w*/*v*) crystal violet followed by incubation at room temperature for 20 min. Excess stains were rinsed off by thorough washing with deionized water and plates were fixed with 200 µL of 30% (*v*/*v*) acetic acid. Blank control (TSB + extract), growth control (cells + TSB), and media control (only TSB) were included in the assay. Optical densities (OD) of stained adherent bacteria were measured at 590 nm using a microplate reader (Infinite M200 PRO, Tecan Group Ltd., Männedorf, Switzerland). All tests were performed in triplicate and repeated twice. The percentage of biofilm inhibition was calculated using the following formula (Equation (2)):

% of biofilm inhibition = [OD growth control − OD sample/OD growth control] × 100(2)

### 3.8. Data Analysis 

Graphs were constructed using average data from triplicate readings of all experimental trials. Statistical significance was calculated using one-way ANOVA at 95% confidence interval followed by Dunnett’s test and/or Tukey test for multiple means comparison. All data were analyzed using GraphPad Prism 8.0.1 Software (GraphPad Software Inc., San Diego, CA, USA).

## 4. Conclusions

The anti-infective efficacy of the methanolic extract and subsequent partition fractions from *P. huayllabambana* fruits have been established as evident in the results obtained from the antibacterial assays. The GC–MS analysis of the n-hexane fraction led to the identification of 11 potentially bioactive components. These phytoconstituents (individually or in interactions with other components) probably act by interfering with bacterial biofilm formation, H^+^-ATPase-mediated proton pumping, and cell membrane integrity. The findings provided a baseline to consider the extracts from *P. huayllabambana* fruits, in particular the n-hexane fraction, in the control of drug-resistant infectious diseases. More investigations are required to isolate, characterize, and evaluate the biological properties of the identified bioactive components. 

## Figures and Tables

**Table 1:** Identified components from the n-hexane fraction of *P. huayllabambana* by gas chromatography–mass spectrometry (GC–MS) analysis.

PK	RT (min)	Chemical Formula	Compounds	Area%	Match % **
1	23.503	C_18_H_36_O	Hexahydrofarnesyl acetone	0.9	90.2
2	23.89	C_16_H_22_O_4_	Diisobutyl phthalate	0.69	85.7
3	25.198	C_17_H_34_O_2_	Methyl palmitate	3.05	92.6
4	25.967	C_16_H_32_O_2_	palmitic acid	11.41	93.6
5	28.375	C_19_H_34_O_2_	Methyl linoleate	1.97	90.4
6	28.507	C_19_H_36_O_2_	Methyl oleate	5.36	92.4
7	28.682	C_20_H_40_O	Phytol	1.96	89.5
8	29.001	C_19_H_38_O_2_	Methyl stearate	1.57	88.1
9	29.314	C_18_H_34_O_2_	Oleic acid	46.55	92.4
10	29.695	C_18_H_34_O_2_	Octadeca-11-enoic acid	7.79	87.4
11	46.384	C_29_H_50_O	beta-Sitosterol	8.3	87.3

PK: number of peaks identified. RT: retention time of the chromatogram (in minutes). ** The significance of the similarity percentage was ≥85% [23].

**Table 2:** Minimal inhibitory concentrations (MICs) of the crude methanolic extract and subsequent partition fractions from *P. huayllabambana* fruits against studied bacterial strains.

Bacteria Strains		MICs (mg/mL) of Crude MeOH Extract and Fractions * of *P. huayllabambana*					METH	STR
		MeOH	n-Hex	DCM	EA	n-BuOH		
Gram-negative	*S. enterica*	0.125	0.25	0.25	0.5	1	0.064	0.004
	*E. cloacae*	1	1	>1	1	>1	>0.256	0.064
	*K. pneumoniae*	1	1	>1	1	>1	>0.256	>0.256
Gram-positive	*S. aureus*	0.5	0.5	1	1	>1	0.125	0.004
	*E. faecalis*	0.25	0.5	0.5	1	>1	0.032	0.125

* Each fraction tested in triplicate. MIC: minimal inhibitory concentration; MeOH: methanol, n-Hex: hexane, DCM: dichloromethane, EA: ethyl acetate, n-BuOH: n-Butanol. Positive controls: methicillin (METH) and streptomycin (STR).
